# Optimizing perovskite solar cell interfaces with molecular bridges

**DOI:** 10.1093/nsr/nwaf478

**Published:** 2025-11-04

**Authors:** Alexandra Ramadan

**Affiliations:** School of Mathematical and Physical Sciences, University of Sheffield, UK

The performance of perovskite solar cells is limited by non-radiative recombination which occurs both in the bulk and at the interfaces with other layers in solar cell devices [1]. These losses can be caused by improper energy level alignment at these interfaces, and/or by defects which can ‘trap’ carriers thus resulting in non-radiative recombination. Whether at the interface or in the bulk, the key to demonstrating high-performance perovskite solar cell devices lies in both the device design and fabrication [2]. Charge transport layers must be selected which reduce the energy barrier to carrier transport [3]. Concomitant fabrication of the layers within the device must be optimized to minimize structural defects and phase impurities.

**Figure 1.**
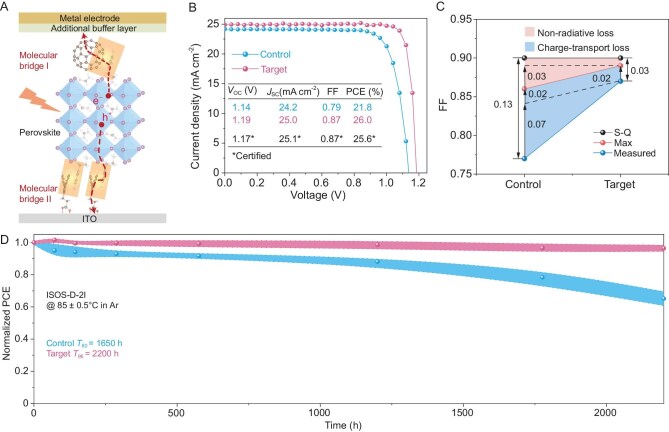
(A) Schematic of dual molecular bridge strategy implemented in perovskite solar cells. (B) Champion current density-voltage (*J-V*) curves under AM 1.5 G simulated solar illumination. (C) Analysis of fill factor (FF) losses including the Shockley–Queisser (S-Q) maximum (max) and measured fill factor. (D) Long-term stability under thermal tracking. Adapted with permission from Ref. [4].

Cheng and co-workers have developed a ‘molecular bridge’ strategy (Fig. 1A) to bind the perovskite and charge transport layer interfaces, reducing non-radiative recombination and simultaneously improving carrier transport across the device [4]. Their approach possesses the benefits of interfacial passivation, enhancing energy level alignment and reducing non-radiative recombination without impacting carrier transport.

The authors designed their molecular bridges using an additive molecule introduced into the perovskite layer, where one end of the molecule binds to vacancies on the perovskite surface whilst the other binds to the surface of the charge transport layer. Density functional theory was used to screen a range of candidates identifying 4-fluoro-phenethylammonium formate (4-F-PEA-Fa), as it possesses multiple chemical moieties which facilitate the formation of a molecular bridge between the perovskite and C_60_ electron transporting layer. To enhance binding of their bridge at the hole transporting layer interface the authors selected [2–10 (7H-dibenzo[c, g]carbazol-7-yl)ethyl]phosphonic acid (DBZ-2PACz) as their hole transport layer. Its incorporation has the dual benefit of improved energy level alignment with the perovskite valence band, and a strong interaction with the 4-F-PEA-Fa bridging molecule through π-π interactions, together suppressing carrier transport losses at this interface.

A benefit of this strategy is its ease of implementation, 4-F-PEA-Fa is introduced into the perovskite precursor solution migrating to the surface and subsequently forming the molecular bridge. Its inclusion also enhances crystallinity, grain structure, and carrier transport resulting in improvements to device performance and stability. Devices incorporating the molecular bridges achieved an impressive champion power conversion efficiency of 26% (Fig. 1B) and a fill factor of 0.87. For the photovoltaic bandgap investigated (1.57 eV), this fill factor (Fig. 1C) represents 97% of that achievable by the detailed balance limit—among the best reported. Furthermore, the large device data set (80 control and 700 target devices) indicates that this approach is consistent and reproducible. The molecular bridge strategy has a dual benefit of enhancing stability alongside efficiency. Investigating a range of different aging conditions, the molecular bridges were found to improve both thermal (Fig. 1D) and operational stability with a T_90_ of 973 h for ISOS-L-1 testing in nitrogen (compared to a T_90_ of 458 h for their control).

Cheng and co-workers’ demonstration of a dual molecular bridge strategy results in excellent solar cell performance, highlighting the importance of interfacial engineering and the potential of molecular bridge strategies to produce reproducible state-of-the-art device performances.
